# Variability of Urinary Concentrations of Bisphenol A in Spot Samples, First Morning Voids, and 24-Hour Collections

**DOI:** 10.1289/ehp.1002701

**Published:** 2011-03-15

**Authors:** Xiaoyun Ye, Lee-Yang Wong, Amber M. Bishop, Antonia M. Calafat

**Affiliations:** 1 Division of Laboratory Sciences, National Center for Environmental Health, Centers for Disease Control and Prevention, Atlanta, Georgia, USA; 2 Battelle Memorial Institute, Atlanta, Georgia, USA

**Keywords:** biomonitoring, bisphenol A, BPA, exposure, human, urine, variability

## Abstract

Background: Human exposure to bisphenol A (BPA) is widespread. After exposure, BPA is rapidly metabolized and eliminated in urine. Therefore, there is considerable within-person and between-person variability of BPA concentrations in spot urine samples. However, no information exists on the within-day variability of urinary BPA concentrations.

Objectives: We examined the between-person and within-person and between-day and within-day variability in the urinary BPA concentrations of eight adults who collected all voids for 1 week to investigate the impact of sampling strategy in the exposure assessment of BPA using spot, first morning, or 24-hr urine collections.

Methods: We determined the urinary concentrations of BPA using on-line solid-phase extraction coupled to isotope dilution high-performance liquid chromatography/tandem mass spectrometry.

Results: The between-day and within-person variability was the primary contributor to the total variance both for first morning voids (77%) and 24-hr urine collections (88%). For the spot collections, we observed considerable within-day variance (70%), which outweighed the between-person (9%) and between-day and within-person (21%) variances.

Conclusions: Regardless of the type of void (spot, first morning, 24-hr collection), urinary BPA concentrations for a given adult changed considerably—both within a day and for the 7 days of the study period. Single 24-hr urine collections accurately reflect daily exposure but can misrepresent variability in daily exposures over time. Of interest, when the population investigated is sufficiently large and samples are randomly collected relative to meal ingestion times and bladder emptying times, the single spot–sampling approach may adequately reflect the average exposure of the population to BPA.

Bisphenol A (BPA) is one of the highest-production-volume chemicals, with > 6 billion pounds produced worldwide each year (Vandenberg et al. 2010). BPA is used primarily to manufacture polycarbonate plastic and epoxy resins, which can be used in impact-resistant safety equipment and baby bottles, as protective coatings in metal food containers, and as composites and sealants in dentistry (European Union 2003; National Toxicology Program 2008). BPA can also be used in the processing of polyvinyl chloride plastic and thermal paper (National Toxicology Program 2008).

Because of its extensive use, human exposure to BPA is widespread. Data from the 2003–2004 National Health and Nutrition Examination Survey (NHANES) conducted by the Centers for Disease Control and Prevention (CDC) showed that 92.6% of individuals sampled among the U.S. general population had detectable levels of BPA in their urine (Calafat et al. 2008). Results from many other studies also have shown BPA exposure in different population groups (Becker et al. 2009; Braun et al. 2009; Calafat et al. 2008; He et al. 2009; Health Canada 2010; Lee et al. 2008; Matsumoto et al. 2003; Teitelbaum et al. 2008; Völkel et al. 2008, 2011; Wolff et al. 2007; Yang et al. 2009; Ye et al. 2005b).

BPA exposure is thought to result primarily from ingesting food (World Health Organization 2010). Upon exposure, BPA is rapidly metabolized and eliminated in urine. Metabolism includes phase II biotransformations, which result in the formation of conjugated species (e.g., glucuronides and sulfates). The total (free plus conjugated) urinary concentration of BPA is a valid biomarker of exposure to BPA (Calafat et al. 2008), and several biomonitoring efforts have evaluated BPA exposure by using spot urine specimens (Calafat et al. 2005, 2008; CDC 2011; Health Canada 2010; Matsumoto et al. 2003; Yang et al. 2003).

Because of the short half-life of BPA (about 6 hr) (Völkel et al. 2002), spot urinary BPA concentrations primarily reflect exposure within a relatively short period preceding urine collection (Koch and Calafat 2009). Furthermore, background exposure to BPA is likely to be episodic (e.g., ingestion of meals) and variable in magnitude. As a result, there is considerable within-person and between-person temporal variability of BPA concentrations in spot urine samples (Arakawa et al. 2004; Mahalingaiah et al. 2008; Nepomnaschy et al. 2009; Teitelbaum et al. 2008), and hourly variability of BPA urinary concentrations can be expected. To the best of our knowledge, no data are available to address this within-day BPA variability in spot urine specimens. Furthermore, because diet, which often changes daily, is thought to be the main pathway of exposure to BPA (World Health Organization 2010), data on the variability of urinary BPA concentrations measured from 24-hr and first morning voids would also be of scientific interest. However, to date, no studies have addressed either within-day variability or variability in BPA urinary concentrations of spot samples, first morning voids, or 24-hr collections obtained from the same person. To address these data gaps, we present here the variability in BPA urinary concentrations from eight adults with no known occupational BPA exposure who collected a total of 427 urine specimens for 7 consecutive days while performing their regular activities.

## Materials and Methods

*Urine collection.* From October through November 2005, a group of four male and four female CDC employees in Atlanta, Georgia, provided urine samples. The group participated in a study designed to examine temporal variability in the urinary concentrations of polycyclic aromatic hydrocarbon (PAH) metabolites (Li et al. 2010). The study participants were nonsmoking volunteers between 26 and 58 years of age who had no documented occupational PAH or BPA exposure. The institutional review board of CDC approved the study, and all participants signed an informed consent.

For a 1-week study period, the eight participants collected a total of 427 samples, including 56 first morning voids, and missed collecting 23 samples [see Supplemental Material, Table 1 (doi:10.1289/ehp.1002701)]. Each participant collected every urine void in a commercial, nonvinyl, nonpolycarbonate plastic specimen collection cup. After recording the volume of each void, the urine was decanted in a prelabeled, sterile urine cup and stored in an ice cooler. The urine samples were retrieved from participants daily (or after the weekend), aliquoted into polypropylene cryovials or glass jars, and stored at –70°C until analysis. During the study week, participants also were asked to record food and drink intake, medications consumed (if any), driving (all participants commuted by car 10–50 miles during the work week), and other activities.

**Table 1:** BPA urinary geometric mean (GM) concentrations for all spot urine samples, first morning voids, and simulated 24-hr urine samples.*a*

Table 1. BPA urinary geometric mean (GM) concentrations for all spot urine samples, first morning voids, and simulated 24-hr urine samples.*a*						
		Frequency of BPA detection (%)		Concentrations (µg/L) [creatinine-corrected (µg/g)]		
Type of void				GM		Median
Spot samples (*n* = 427)		91		2.8 (4.2)		1.7 (2.7)
First morning voids (*n* = 56)		100		2.7 (3.5)		2.3 (2.8)
Simulated 24-hr voids (*n* = 56)		100		2.8 (3.7)		2.4 (3.3)
**a**For comparison purposes, for adults (*n* = 951) from 2003–2004 NHANES (Calafat et al. 2008), the GM and median concentrations were 2.6 µg/L (2.4 µg/g) and 2.7 µg/L (2.4 µg/g), respectively.						

*Analytical method for measuring BPA.* The total BPA concentration was measured using a mass spectrometry method described previously (Ye et al. 2005a). The limit of detection (LOD) was 0.4 µg/L. To ensure data accuracy and precision, each batch of samples included quality control (QC) samples, standards, and reagent blanks. The QC concentrations were evaluated using standard statistical probability rules (Caudill et al. 2008). Urinary creatinine, used to correct the dilution of the urine, was measured at CDC using a Roche Hitachi 912 Chemistry Analyzer (Hitachi, Pleasanton, CA).

*Statistical analysis.* We performed the statistical analyses using SAS software (SAS Institute Inc., Cary, NC). For concentrations below the LOD, we used a value equal to the LOD divided by the square root of 2 (Hornung and Reed 1990). The urinary BPA concentration followed a log-normal distribution. Thus, before statistical analysis, all data were log_10_ transformed.

The first morning void was defined as the first sample collected by each person at or after 5:00 a.m. each day. The simulated 24-hr void concentration was calculated based on the volume-weighted average of all urine samples collected (missed collections were not accounted for) during a 24-hr period starting after midnight. To assess the impact of creatinine adjustment on the total variance when exposure is categorized from the BPA concentrations of spot urine samples, we built three different models. For model A, BPA was not creatinine-corrected (log_10_ noncorrected concentration in micrograms per liter), whereas for model B, BPA was creatinine-corrected to account for urinary dilution (log_10_ creatinine-corrected concentration in micrograms per gram creatinine). Model C adjusted for urine dilution by including creatinine as a model covariate (log_10_-adjusted concentration in micrograms per liter). We ranked these models based on their Akaike information criterion (AIC; Akaike 1974) (the lower the AIC, the better the model). To assess the temporal variability in BPA concentrations of spot samples, first morning voids, and simulated 24-hr voids, we calculated intraclass correlation coefficients (ICCs) using a three-level model. Level 1 is the time (*i*), which is irregular, unequal, and interval nested within the day (level 2, *j* = 7), which is nested within the participants (level 3, *k* = 8). The equation for models A and B was *Y_ijk_* = (*Y*_000_) + (*V*_00_*_k_* + *U*_0_*_jk_* + γ*_ijk_*), where *Y_ijk_* (the dependent variable) is the log_10_ (BPA) (for model A) or log_10_ (creatinine-corrected BPA) (for model B) for participant *k* on day *j* at time *i*. The equation for model C includes creatinine as an additional independent variable, with log_10_ (BPA) as the dependent variable. The intercept Y_000_ is the grand mean (i.e., the average value across all observations), and *V*_00_*_k_*, *U*_0_*_jk_*, and γ*_ijk_* are the random errors for level 3, level 2, and level 1 residual, respectively (Singer 1998). The ICC indicates the temporal reproducibility of repeated measures and is computed by dividing the estimate of the between-subject variance by the estimated total variance. ICC ranges from 0 (poor reproducibility) to 1 (perfect reproducibility).

For the spot urine samples, we also compared the variance apportionment of BPA and creatinine concentrations by constructing a model in which creatinine concentration was the outcome. We also checked the effect of the missed collections on the variation pattern of urinary BPA in spot samples by comparing the variance apportionment of urinary BPA concentrations in spot samples with and without participant 2, who had the largest number of missed collections [see Supplemental Material, Table 1 (doi:10.1289/ehp.1002701)].

## Results

**Figure 1:**
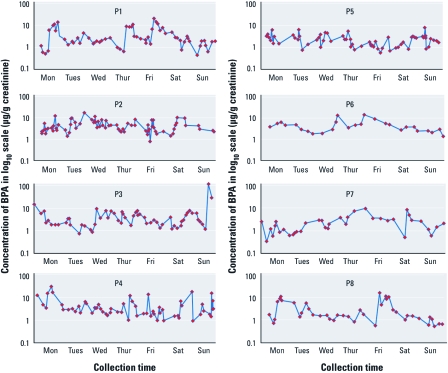
Concentration of BPA in log_10_ scale (µg/g creatinine) for all of the spot urine samples collected from eight participants over 1 week.

Table 1 lists the noncorrected and creatinine-corrected geometric mean (GM) and median urinary BPA concentrations of the spot, first morning, and simulated 24-hr urine voids, along with the frequency of BPA detection. The creatinine-corrected GMs of spot urine samples, first morning voids, and simulated 24-hr urine samples ranged from 3.5 µg/g to 4.2 µg/g. The creatinine-corrected median BPA concentrations ranged from 2.7 µg/g to 3.3 µg/g. We detected BPA in 91% of the spot samples. Table 2 lists the daily average BPA concentrations (in micrograms per gram creatinine) estimated from each participant’s spot, first morning, and simulated 24-hr urine voids during the study week. We observed that the daily urinary BPA concentrations were occasionally normally distributed, but not consistently for any participant [see Supplemental Material, Table 2 (doi:10.1289/ehp.1002701)]. Figure 1 shows the BPA concentrations [in log_10_ scale (micrograms per gram creatinine)] for all of the spot urine samples collected by each of the eight participants over 1 week. All participants collected urine samples from Monday through Sunday except participant 3, who collected the urine from Saturday to the following Friday. No clear exposure pattern of BPA was observed throughout the 7 days of collection for the eight participants. However, for each person, the urinary BPA concentrations could vary up to two orders of magnitude within a given day. For example, for participant 3 on Sunday, BPA urinary concentrations in spot samples varied from 1.3 µg/g to 117.7 µg/g (Figure 1). As a result, the within-day coefficient of variation (CV%) from spot urine measurements ranged from 9% to 177%. We also observed considerable variation in the within-person and within-day BPA concentrations for the first morning and simulated 24-hr urine voids (Table 2). In any given day, the BPA urinary concentrations from first morning and simulated 24-hr urine collections could be rather different, even for the same person [e.g., participant 8 on Friday: 0.6 µg/g (first morning) and 6.1 µg/g (24-hr collection)]. Table 2 also lists the mean creatinine-corrected urinary BPA concentrations of spot, first morning, and simulated 24-hr voids from each participant averaged over the entire study period. The within-person variation CV% in BPA urinary concentrations ranged from 63% to 235% (spot collections), 53% to 120% (first morning voids), and 25% to 85% (simulated 24-hr voids). For each participant, we also calculated the daily total BPA exposure (in micrograms per day) during the study period by summing the amounts of BPA [in micrograms, obtained by multiplying the BPA concentrations (in micrograms per liter) in each spot urine sample by the spot urine volume (in liters)] excreted in one given day (Table 3). Of interest, we observed considerable within-person and between-days variation for BPA total daily exposure, with CV% ranging from 23% (participant 5) to 97% (participant 3).

**Table 2:** BPA concentrations of spot urines, first morning voids, and simulated 24-hr urine voids from each participant during the study week.*a,b*

Table 2. BPA concentrations of spot urines, first morning voids, and simulated 24-hr urine voids from each participant during the study week.*a,b*																		
				BPA urinary concentrations of participant (µg/g creatinine)														
Week day		Void type		P1		P2		P3		P4		P5		P6		P7		P8
Monday		Spot collection		5.5 (4.9)		3.8 (2.5)		5.0 (4.3)		14.2 (8.9)		3.1 (1.5)		5.0 (0.9)		1.4 (0.9)		5.6 (0.8)
		First morning void		1.2		2.0		6.0		5.1		3.2		3.7		0.3		1.7
		24-hr void		4.8		4.0		4.4		11.8		2.9		4.8		1.5		3.7
Tuesday		Spot collection		2.3 (1)		6.4 (4.7)		2.2 (0.9)		3.7 (1.3)		2.9 (1.7)		2.9 (1)		1.1 (0.6)		3.4 (1.8)
		First morning void		1.4		2.7		2.4		3.0		3.7		4.6		0.7		6.0
		24-hr void		2.5		5.9		2.1		4.0		2.8		3.1		1.1		4.0
Wednesday		Spot collection		2.1 (0.4)		6.3 (2.3)		4.6 (3.2)		3.2 (1.1)		2.9 (1.2)		6.1 (4.5)		2.1 (0.7)		1.4 (0.3)
		First morning void		1.5		8.3		0.9		2.2		2.2		1.9		3.1		1.6
		24-hr urine		2.2		5.9		3.8		2.8		2.7		3.7		2.5		1.5
Thursday		Spot collection		5.3 (4.1)		4.1 (1.8)		5.0 (2.1)		4.5 (3.4)		2.2 (1.4)		6.8 (4.3)		5.4 (2.8)		1.6 (0.7)
		First morning void		1.0		3.0		3.0		2.4		3.2		3.4		2.0		1.4
		24-hr void		3.9		3.5		4.1		3.9		2.4		5.0		6.6		1.6
Friday		Spot collection		7.2 (6)		3.4 (2.1)		3.2 (1.4)		3.4 (3.5)		1.4 (0.6)		6.4 (1.8)		3.4 (0.3)		8.1 (5.1)
		First morning void		2.2		2.5		3.9		2.0		1.7		9.0		3.6		0.6
		24-hr void		7.0		2.7		3.0		2.9		1.7		6.8		3.3		6.1
Saturday		Spot collection		2.7 (1.2)		5.1 (3.1)		3.6 (2.3)		2.8 (2.2)		1.7 (1)		3.2 (3.1)		4.1 (2.7)		1.5 (0.4)
		First morning void		5.0		1.5		4.1		1.6		1.0		2.7		0.5		1.1
		24-hr void		3.2		3.9		4.0		2.4		1.4		3.1		3.9		1.4
Sunday		Spot collection		1.6 (0.6)		3.0 (0.8)		20.1 (35.5)		5.6 (5.6)		2.7 (1.7)		2.2 (0.6)		1.6 (0.7)		0.8 (0.3)
		First morning void		0.4		4.2		6.1		1.9		3.2		2.4		1.2		0.6
		24-hr void		1.3		3.4		16.6		3.7		2.5		2.3		1.9		0.7
Monday–Sunday*c*		Spot collection		4.0 (4.2)		4.7 (3.0)		6.4 (15.1)		5.0 (5.3)		2.4 (1.5)		4.6 (3.1)		2.6 (2.2)		3.5 (3.8)
		First morning void		2.0 (2.4)		3.9 (2.1)		3.2 (2.2)		1.9 (1.4)		4.9 (3.2)		5.3 (2.8)		2.9 (2.8)		3.9 (4.4)
		24-hr void		3.6 (1.8)		4.0 (1.0)		5.4 (4.6)		4.5 (3.0)		2.4 (0.5)		4.1 (1.4)		3.0 (1.8)		2.7 (1.8)
**a**For the spot samples, the values are the arithmetic mean concentrations of all samples collected daily from each participant. **b**The number in parenthesis by the mean concentration of the spot collections is the SD. **c**For Monday through Sunday, the values are the mean ± SD concentrations of all spot, first morning voids, or 24-hr urine samples collected from each participant during 1 week.																		

**Table 3:** Total daily exposure (micrograms) to BPA calculated from the BPA concentrations in the spot urine samples collected from eight participants during the study week.*a*

Table 3. Total daily exposure (micrograms) to BPA calculated from the BPA concentrations in the spot urine samples collected from eight participants during the study week.*a*																
		Total daily exposure of participant (µg)														
Day		P1		P2		P3		P4		P5		P6		P7		P8
Monday		5.9		3.3		4.4		9.5		4.1		7.6		3.6		4.4
Tuesday		3.1		4.3		1.7		7.0		5.6		5.2		1.8		6.5
Wednesday		2.8		5.2		3.9		3.6		5.8		6.1		3.3		1.9
Thursday		5.5		4.7		4.0		4.6		5.8		8.1		13.0		2.3
Friday		8.7		2.5		3.0		3.8		3.4		11.3		5.2		11.0
Saturday		3.9		3.7		4.6		2.0		3.2		4.9		4.4		2.0
Sunday		1.5		1.2		19.7		4.0		4.5		3.8		4.5		1.1
Mean																
(Monday–Sunday)*a*		4.5 ± 2.2		3.5 ± 1.3		5.9 ± 5.7		4.9 ± 2.3		4.6 ± 1.1		6.7 ± 2.3		5.1 ± 3.4		4.2 ± 3.2
**a**Mean ± SD of the total daily exposure of BPA from Monday through Sunday.																

We used three different models to evaluate how accounting for urine dilution might affect BPA concentrations in spot samples (Table 4). Model A, without creatinine correction for urinary dilution, gave the highest AIC value (480.1), which indicated the worst fit of the three models. AIC was similar for the models that accounted for the dilution of the urine either by dividing the BPA concentrations by the creatinine concentration (model B, AIC = 268.8) or by using creatinine as a covariate in the model (model C, AIC = 288.6). We did not observe a clear pattern of creatinine excretion among the participants [Supplemental Material, Figure 1 (doi:10.1289/ehp.1002701)]. In addition, the results of the model in which creatinine concentration was the outcome suggested that the variance patterns of creatinine and BPA concentrations were similar [see Supplemental Material, Table 3 (doi:10.1289/ehp.1002701)]. However, the differences in urine dilution still explained some of the observed variance in BPA concentrations of the spot samples, because when we accounted for urine dilution, the model fits improved (models B and C vs. A). Therefore, for all subsequent variance calculations, we used the creatinine-corrected concentrations (in micrograms per gram creatinine).

**Table 4:** Effect of creatinine correction in the variance apportionment for the urinary concentrations of BPA in spot urine samples collected from eight persons over a 1-week period.

Table 4. Effect of creatinine correction in the variance apportionment for the urinary concentrations of BPA in spot urine samples collected from eight persons over a 1-week period.												
		Model A*a*				Model B*b*				Model C*c*		
Variance parameter		Variance component		Percentage of total variance		Variance component		Percentage of total variance		Variance component		Percentage of total variance
Between persons		0.0281		14		0.0117		9		0.0090		7
Within person/between days		0.0244		12		0.0269		21		0.0264		20
Within person/within day		0.1536		74		0.0902		70		0.0945		73
**a**Model A: log_10_ of BPA concentration as the outcome without creatinine adjustment (AIC = 480.1).** b**Model B: log_10_ of creatinine-corrected BPA concentration as the outcome (AIC = 268.8). **c**Model C: log_10_ of BPA concentration as the outcome using creatinine as covariate (AIC = 288.6).												

Table 5 lists the contribution to the total variance of the log-transformed creatinine-corrected concentration of BPA between persons and within persons in 7 consecutive days of sampling for all spot, first morning, and simulated 24-hr voids. For the spot collections, the within-day variation was the main contributor (70%) to the variance, followed by between-day (21%) and between-person (9%) variability. Results were comparable when participant 2, who missed 14 urine collections, was excluded from the analysis (Table 5). For the first morning voids, the within-person variability (77%) outweighed the between-participants variability (23%). Similarly, for the simulated 24-hr urine collections, the within-participants’ variance contribution (88%) was higher than the between-person variance (12%).

**Table 5:** The variance apportionment of the log-transformed creatinine-corrected concentration of BPA in urine samples collected from eight people over a 1-week period.

Table 5. The variance apportionment of the log-transformed creatinine-corrected concentration of BPA in urine samples collected from eight people over a 1-week period.												
		Spot urine collection*a*				First morning urine voids				24-hr urine void		
Variance parameter		Variance component		Percentage of total variance		Variance component		Percentage of total variance		Variance component		Percentage of total variance
Between persons		0.0117		9		0.0234		23		0.0070		12
Within person/between days)		0.0267		21		0.0796		77		0.0521		88
Within person/within day)		0.0902		70								
**a**The variance component (percentage of total variance) of urinary concentrations of BPA in spot urine samples without participant 2 who had the most missing spot samples was 0.0103 (7%; between persons), 0.0296 (22%; within person and between days), and 0.0973 (71%; within person and within day).												

## Discussion

The BPA creatinine-corrected concentrations from the spot urine samples collected for this study were of the same order of magnitude as those reported for 2003–2004 NHANES adults (Calafat et al. 2008), with similar median and GM concentrations. Furthermore, the BPA detection frequency in the spot urine samples collected from our study population (91%) was similar to the frequency of detection from NHANES 2003–2004 (92.6%), which was also based on spot sample concentrations. Although our study population was limited in size, our findings suggest that exposure to BPA, estimated from the urinary concentrations in spot samples from the adults we examined, fell within the reference ranges reported for the general U.S. adult population.

Temporal variability in urinary BPA concentrations is likely due to changing exposure throughout the day and across days, driven by the diet and other lifestyle choices of the person. A few studies have investigated the temporal variability of urinary BPA (Aikawa et al. 2004; Braun et al. 2011; Mahalingaiah et al. 2008; Nepomnaschy et al. 2009; Teitelbaum et al. 2008). Large within-person variability appeared in urinary BPA measurements of three first morning voids collected from 60 premenopausal women during approximately 4 weeks (Nepomnaschy et al. 2009). Other studies also indicated the temporal within-person and between-person variance in urinary BPA concentrations from different populations of children and adults over periods ranging from days to months (Aikawa et al. 2004; Braun et al. 2011; Mahalingaiah et al. 2008; Teitelbaum et al. 2008). We also observed relatively low between-person ICCs (0.09–0.23) for the creatinine-corrected BPA concentrations of spot, first morning, and simulated 24-hr urine collections. This finding was in agreement with the results reported from two previous studies of 35 children who collected up to 159 spot urine samples for > 6 months (ICC = 0.35) (Teitelbaum et al. 2008) and of 389 women who provided three spot samples during pregnancy and at birth (ICC = 0.11) (Braun et al. 2011). These results suggest a high within-person variation and a rather low reproducibility of the BPA concentrations among repeated urine collections from the same person. Similar within-person and within-day variance has been observed for other compounds such as some PAHs (Li et al. 2010) and di(2-ethylhexyl) phthalate (DEHP) (Preau et al. 2010).

As stated, human BPA exposure occurs mainly through diet (World Health Organization 2010), which may be highly variable among adults. We observed rather high between-day variance for each participant for the first morning (77%) and simulated 24-hr (88%) urine voids. We also observed a high within-person and between-day variance in the total daily exposure to BPA estimated from the BPA concentrations in spot samples. Of interest, the U.S. Environmental Protection Agency (1996) has recommended 24-hr urine collection for evaluating exposure to pesticides and other toxic chemicals excreted primarily through urine. Similarly, many epidemiologic studies have used the first morning urine void because of its correlation with 24-hr urine (Kissel et al. 2005; Scher et al. 2007). Still, depending on the exposure source of the target compound, the considerably high between-day variance of the 24-hr urine collections and the high variance of the total daily exposure mean that collecting 24-hr urine voids from a person only once might not be the best approach to estimate exposure of this person throughout a period of days, weeks, or months. Furthermore, given the considerable differences in BPA urinary concentrations between the first morning and 24-hr voids that we observed for some participants on any given day, first morning voids may not be good surrogates for 24-hr collections, at least in specific cases (e.g., a first morning void collected after having conducted an activity associated with exposure to BPA, such as consuming a BPA-rich meal, the night before).

The mean urinary BPA concentrations calculated from the spot urine samples collected by each participant during 1 week were closer to the mean concentrations of 24-hr collections than to the first morning void concentrations. However, we observed notable within-day variations of BPA concentrations in spot urine samples collected from the same participant. This high within-day variability from spot urine samples was not unexpected because of the nature of the exposure (e.g., episodic ingestion of meals) and rapid urinary elimination of BPA. Of interest, for the spot collections, was that the within-day variability was the major contributor to the total variance and outweighed both the between-person and the between-day and within-person contributions. Although some persons missed collecting several voids, we did not observe much difference in the variance pattern of the BPA concentrations from spot urine samples based on a sensitivity analysis in which we excluded participant 2 who had the most missing samples. Nonetheless, our results suggest that for a given participant, the urinary concentrations of BPA can change considerably throughout the day. Thus, at a minimum, we recommend recording the time of day of urine collection and of the last urination. Depending on the diet, which for an adult normally varies not only from day to day, but also from meal to meal, the timing of sample collection relative to the time of food consumption and previous bladder-voiding times has a direct impact on the estimated exposure to BPA based on the concentrations of BPA in spot specimens. More important, our findings are in close agreement with those reported for DEHP, another compound for which diet is a main exposure source, where the within-day variance was the major contributor of the total variance for spot urine specimens (Preau et al. 2010).

Our findings reemphasize the importance of sampling strategies during the design of an epidemiological study. Such strategies should be tailored to the study population. For eample, age that may also affect exposure to environmental chemicals, including BPA. Behavior, diet, and potentially age-related differences in metabolism, among other factors, likely contribute to BPA exposure. Thus, some of the findings we report for this group of eight adults may not apply to children, pregnant women, and other adult groups of different ages. Even though timing and frequency of the sampling should depend mainly on the route of uptake of the parent compound and the half-life of the excreted metabolites, our data suggest that when multiple collections of spot urine samples over a period of time (e.g., days, weeks, months) are logistically and economically possible, the samples should be randomly collected relative to the times a meal is ingested and and the times a bladder is emptied.

## Conclusions

Exposure variability over time is an important factor for interpreting BPA biomonitoring data for risk assessment (Vandenberg et al. 2010). We present here the variability in the urinary concentrations of BPA from samples collected from eight adults for a period of 7 consecutive days. Our data suggest that the within-person and between-day variability was notably higher than the between-person contribution for the urinary BPA concentrations obtained from first morning voids and 24-hr urine collections. More important, we observed a significant within-day variance in BPA urinary concentrations of spot samples collected from the same person. Single 24-hr urine collections accurately reflect daily exposure but cannot represent variability in daily exposures over time. Therefore, collecting one 24-hr urine sample per person will not eliminate the potential for exposure misclassification, because such an approach would not account for daily variability in exposure. Single spot samples, including first morning voids, will not eliminate the potential for a person’s exposure misclassification either. However, when samples are collected from a large number of persons (e.g., population surveys like NHANES) and randomly collected relative to meal ingestion times and bladder emptying times, the single spot–sampling approach may adequately reflect the average exposure of a population to BPA. Despite the limitations associated with the temporality of urinary measures of BPA and other nonpersistent chemicals, biomonitoring measures will considerably strengthen an exposure assessment.

## Supplemental Material

(96 KB) PDF
