# Supplementary material for: Variability of Urinary Concentrations of Bisphenol A in Spot Samples, First Morning Voids, and 24-Hour Collections
Source: Environ Health Perspect. 2011 Mar 15;119(7):983–8. doi: 10.1289/ehp.1002701 (PMC3223007; doi:10.1289/ehp.1002701)

Supplemental Material

Variability of Urinary Concentrations of Bisphenol A in Spot Samples, First-morning Voids, and 24-Hour Collections

Xiaoyun Ye[1]*, Lee-Yang Wong[1], Amber M. Bishop[2], and Antonia M. Calafat[1]

[1]Division of Laboratory Sciences, National Center for Environmental Health,

Centers for Disease Control and Prevention, Atlanta, GA 30341

4770 Buford Hwy, Mailstop F53,

Atlanta, Georgia 30341, USA

[2] Battelle Memorial Institute

2987 Clairmont Rd St 450

Atlanta, Georgia 30329, USA

Supplemental Material, Table 1. Demographic characteristics of the study participants and logistics of sample collection (The numbers in parentheses indicate the number of missed collections).

ID	Age	Sex	Sampling period (2005)	Total# samples collected	Total# samples missed	Number of samples collected						
						Mon	Tues	Wed	Thur	Fri	Sat	Sun
P1	31	F	11/14–11/20	63	0	10	7	8	9	11	10	8
P2	25	F	11/14–11/20	74	14	15	10 (3)	14 (1)	10 (1)	13 (2)	8 (3)	4 (4)
P3	59	F	10/29–11/04	60	1	8	8	8	10	9	10	9 (1)
P4	27	F	10/31–11/06	61	5	6 (2)	8	12	8	10	7	11 (3)
P5	34	M	11/14–11/20	64	1	8 (1)	7	8	9	10	9	13
P6	26	M	10/31–11/06	27	0	4	4	4	4	3	4	4
P7	32	M	11/14–11/20	37	2	8	6	5	5 (1)	3 (1)	5	5
P8	32	M	11/14–11/20	41	0	7	6	5	5	7	5	6
			TOTAL:	427	23							

Supplemental Material, Table 2. Normal daily distribution of the urinary concentrations of BPA from spot samples collected per participant based on Shapiro-Wilk statistics.[a]

Participant	Day of the week						
	Mon	Tues	Wed	Thur	Fri	Sat	Sun
P1	N	Y	Y	N	Y	N	N
P2	N	Y	N	N	N	Y	N
P3	N	Y	N	N	N	Y	N
P4	Y	N	N	Y	N	Y	N
P5	Y	N	Y	N	N	N	Y
P6	N	Y	Y	Y	Y	Y	Y
P7	N	Y	N	N	Y	Y	Y
P8	Y	Y	Y	Y	N	Y	Y

[a]Y and N refer to normal and not normal distributions, respectively. Y for P value > 0.05, N for P value < 0.05

Supplemental Material, Table 3. Variance apportionment of log_{10}-transformed creatinine concentration as the outcome in spot urine samples collected from eight persons over a one week period.[a]

Variance parameter	Log_{10}-transformed creatinine concentration as the outcome	
	Variance component	% of total variance
Between persons	0.034	23
Within person/ between days	0.0025	2
Within person/within day	0.1081	75

[a]Akaike information criterion (AIC): 302.9

Supplemental Material, Figure 1. Creatinine concentrations (g/mL) for all of the spot urine samples collected from eight participants over one week.

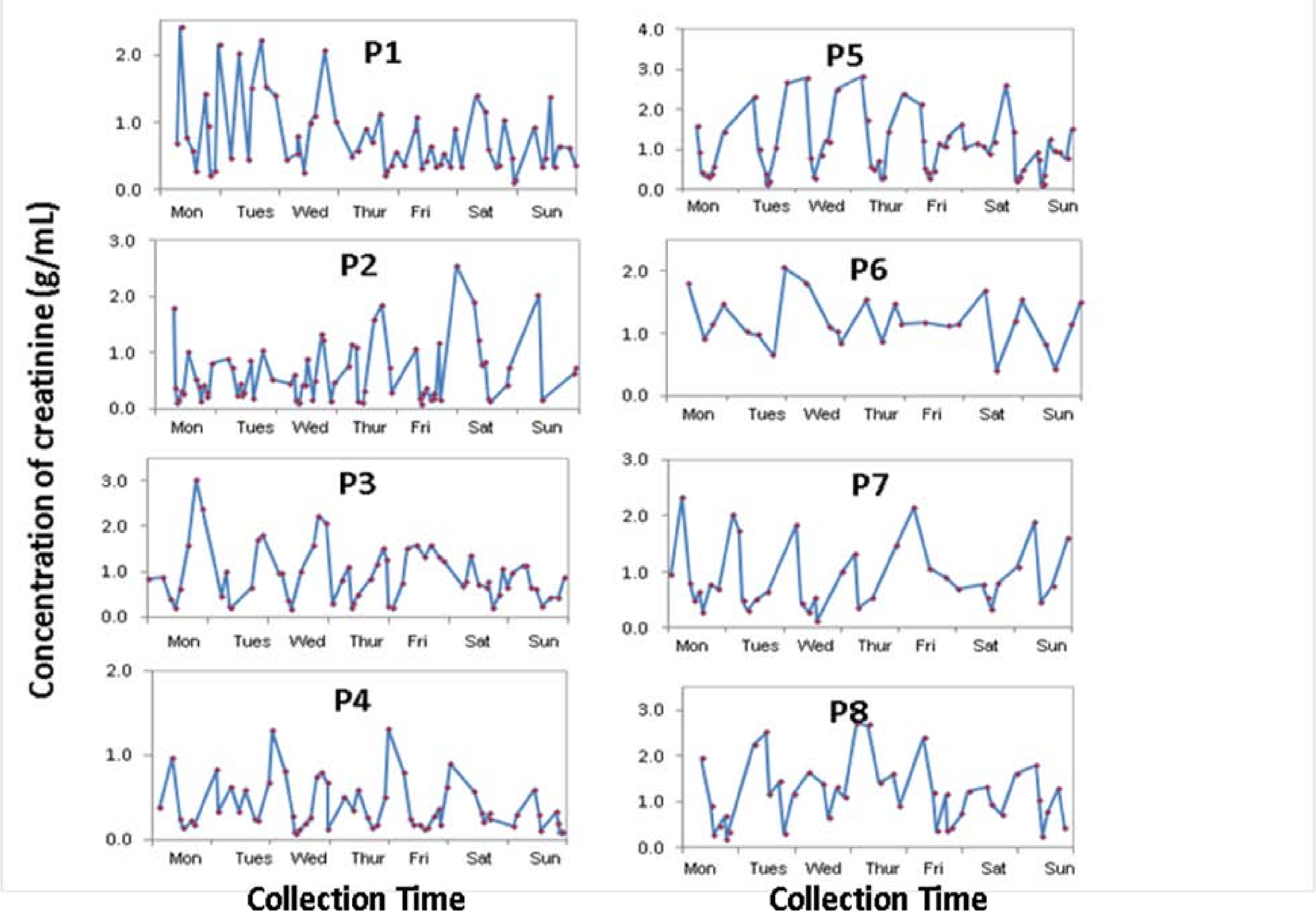

Supplement: (96 KB) PDF [file ehp.1002701.s001.pdf]
